# Medroxyprogesterone acetate alters the vaginal microbiota and microenvironment in women and increases susceptibility to HIV-1 in humanized mice

**DOI:** 10.1242/dmm.039669

**Published:** 2019-10-01

**Authors:** Jocelyn M. Wessels, Julie Lajoie, Maeve I. J. Hay Cooper, Kenneth Omollo, Allison M. Felker, Danielle Vitali, Haley A. Dupont, Philip V. Nguyen, Kristen Mueller, Fatemeh Vahedi, Joshua Kimani, Julius Oyugi, Juliana Cheruiyot, John N. Mungai, Alexandre Deshiere, Michel J. Tremblay, Tony Mazzulli, Jennifer C. Stearns, Ali A. Ashkar, Keith R. Fowke, Michael G. Surette, Charu Kaushic

**Affiliations:** 1 McMaster Immunology Research Centre, Michael G. DeGroote Centre for Learning and Discovery, McMaster University, Hamilton, Ontario L8S 4K1, Canada; 2 Department of Pathology and Molecular Medicine, McMaster University, Hamilton, Ontario L8S 4K1, Canada; 3 Department of Medical Microbiology, University of Manitoba, Winnipeg, Manitoba R3E 0J9, Canada; 4 Department of Medical Microbiology, University of Nairobi, P.O. BOX 30197-00100, Nairobi, Kenya; 5 Kenyan AIDS Control Program, P.O. Box 19361 - 00202, Nairobi, Kenya; 6 Axe des Maladies Infectieuses et Immunitaires, Centre de Recherche du CHU de Québec-Université Laval, Pavillon CHUL, Québec City, Québec G1V 4G2, Canada; 7 Department of Microbiology and Immunology Medical Biology, Université Laval, Québec City, Québec G1V 0A6, Canada; 8 Public Health Laboratories, Public Health Ontario, Toronto, Ontario M5G 1V2, Canada; 9 Mount Sinai Hospital/University Health Network, Department of Microbiology, Toronto, Ontario M5G 1X5, Canada; 10 Department of Laboratory Medicine and Pathobiology, University of Toronto, Toronto, Ontario M5S 1A8, Canada; 11 Department of Medicine, Farncombe Family Digestive Health Institute, McMaster University, Hamilton, Ontario L8S 4K1, Canada; 12 Department of Biochemistry and Biomedical Sciences, McMaster University, Hamilton, Ontario L8S 4K1, Canada; 13 McMaster Institute of Infectious Disease Research, McMaster University, Hamilton, Ontario L8S 4K1, Canada

**Keywords:** DMPA, Glycogen, Amylase, Polymicrobial vaginal microbiota, Humanized mouse

## Abstract

The hormonal contraceptive medroxyprogesterone acetate (MPA) is associated with increased risk of human immunodeficiency virus (HIV), via incompletely understood mechanisms. Increased diversity in the vaginal microbiota modulates genital inflammation and is associated with increased HIV-1 acquisition. However, the effect of MPA on diversity of the vaginal microbiota is relatively unknown. In a cohort of female Kenyan sex workers, negative for sexually transmitted infections (STIs), with Nugent scores <7 (*N*=58 of 370 screened), MPA correlated with significantly increased diversity of the vaginal microbiota as assessed by 16S rRNA gene sequencing. MPA was also significantly associated with decreased levels of estrogen in the plasma, and low vaginal glycogen and α-amylase, factors implicated in vaginal colonization by lactobacilli, bacteria that are believed to protect against STIs. In a humanized mouse model, MPA treatment was associated with low serum estrogen, low glycogen and enhanced HIV-1 susceptibility. The mechanism by which the MPA-mediated changes in the vaginal microbiota may contribute to HIV-1 susceptibility in humans appears to be independent of inflammatory cytokines and/or activated T cells. Altogether, these results suggest MPA-induced hypo-estrogenism may alter key metabolic components that are necessary for vaginal colonization by certain bacterial species including lactobacilli, and allow for greater bacterial diversity in the vaginal microbiota.

This article has an associated First Person interview with the first author of the paper.

## Abstract (precis)

**Summary:** MPA may increase susceptibility to HIV-1 in sex workers through the suppression of endogenous estrogen, reducing vaginal glycogen and α-amylase levels, which increases bacterial diversity, potentially reducing protective bacterial species such as lactobacilli.

## INTRODUCTION

Meta-analyses suggest that the injectable progestin-based contraceptive depot medroxyprogesterone acetate (DMPA) increases heterosexual acquisition of human immunodeficiency virus (HIV-1) 1.4-fold (Polis et al., 2016), and a prospective study reported women using injectable progestins were at 3.5× increased risk of HIV acquisition compared with women not using long-term hormonal contraceptives (Byrne et al., 2016). These statistics are particularly troubling because DMPA is a popular contraceptive in Africa (Ross and Agwanda, 2012), where HIV-1 prevalence is greatest. Studies have suggested several biological mechanisms, including hypo-estrogenism, by which DMPA might enhance susceptibility to HIV-1 and other sexually transmitted infections (STIs) (Hapgood et al., 2018). However, few (Borgdorff et al., 2015; Brooks et al., 2017; Jespers et al., 2017; Kazi et al., 2012; Mitchell et al., 2014; Roxby et al., 2016) examine the relationship between hormonal contraceptives and the vaginal microbiota (VMB), even though diverse VMB, low in *Lactobacillus* species, confers a 4-fold increased risk of HIV-1 (Gosmann et al., 2017). Here, we determined the effect of MPA (the active component of DMPA) on several factors within the vaginal microenvironment of Kenyan sex workers, including diversity of the VMB.

The VMB is a bacterial community that lines vaginal epithelial cells (Ravel et al., 2011). Unlike the diverse gut microbiota, the VMB is generally low in diversity, and five community state types, including four dominated by a *Lactobacillus* species, have been described (Ravel et al., 2011). Two factors thought to maintain vaginal lactobacilli are glycogen, stored in epithelial cells and available as free glycogen in vaginal fluid (Gregoire et al., 1971; Mirmonsef et al., 2014; Nasioudis et al., 2015), and α-amylase, an enzyme that catabolizes glycogen for use by lactobacilli and other bacteria (Macklaim et al., 2013) as energy. Similar to the gut, the VMB modifies immunity. The VMB alters inflammation in the female genital tract (Anahtar et al., 2015), and is implicated in reproductive health and disease. Several factors including ethnicity (Ravel et al., 2011), STIs (Borgdorff et al., 2014), bacterial vaginosis (BV) (Gajer et al., 2012; Srinivasan et al., 2012) and sex work (Wessels et al., 2017) have been reported to impact VMB composition. This study was designed to examine the effect of hormonal contraceptives on the VMB of healthy, asymptomatic Kenyan sex workers to determine whether DMPA is associated with changes in the VMB and vaginal microenvironment that might impact their susceptibility to HIV-1. Importantly, we chose to exclude women with BV because it is a clinical condition reported to profoundly overshadow the effect of hormones on vaginal biomarkers of microbial health (glycosidases, lectins) in cervico-vaginal lavage (CVL) (Moncla et al., 2016, 2015). Furthermore, we (Wessels et al., 2017) and others (Gajer et al., 2012; Srinivasan et al., 2012) have demonstrated by 16S ribosomal RNA (rRNA) gene sequencing that women diagnosed with BV by Nugent scoring have diverse VMBs, but women with high diversity VMBs do not always have Nugent scores indicative of BV (McKinnon et al., 2019; Wessels et al., 2017). Therefore, because symptomatic BV is a clinical condition that could potentially confound the relationship between DMPA and diversity of the VMB, and alter vaginal biomarkers of microbial health, the present study excluded women with Nugent scores 7-10, which is used to diagnose clinical BV.

Meta-analyses find that DMPA correlates with increased HIV-1 susceptibility in women (Polis et al., 2016), and that women with diverse VMB have increased susceptibility to HIV-1 (Gosmann et al., 2017). Based on these observations, we hypothesized that MPA will increase diversity of the VMB, which in turn will affect HIV-1 risk by impacting inflammatory cytokines and HIV-1 target cells. Here, we examine the effect of MPA on the VMB, vaginal glycogen and vaginal α-amylase in healthy, asymptomatic Kenyan sex workers. We also explore the relationship between bacterial diversity, vaginal cytokines and HIV-1 target cells. Our overall aim was to perform bacterial 16S rRNA gene sequencing of the VMB of sex workers using DMPA, oral contraceptives (OCPs), or not using hormonal contraceptives (NH; proliferative phase of the menstrual cycle), and assess the effect of hormonal contraceptives on the vaginal microenvironment and diversity of the VMB, as they relate to HIV-1 susceptibility. We found that DMPA use was associated with decreased plasma estrogen levels, increased VMB diversity, and that vaginal glycogen and α-amylase were lower in DMPA-treated sex workers. We experimentally recapitulated some of the results of our clinical study in humanized mice, and demonstrated that DMPA enhanced HIV-1 susceptibility. Results suggest MPA-induced hypo-estrogenism alters key metabolic products (i.e. glycogen and α-amylase) that are important for vaginal colonization by protective bacterial species such as lactobacilli, and the change in substrates might allow for greater bacterial diversity. However, contrary to our hypothesis, the mechanism by which DMPA-mediated changes in the vaginal microbiota might affect HIV-1 susceptibility in this cohort of Kenyan sex workers with Nugent scores <7 appears to be independent of inflammatory cytokines and/or activated T cells, unlike results reported in women with BV.

## RESULTS

### DMPA is associated with vaginal microbial diversity in healthy, asymptomatic Kenyan sex workers

**Fig. 1.**
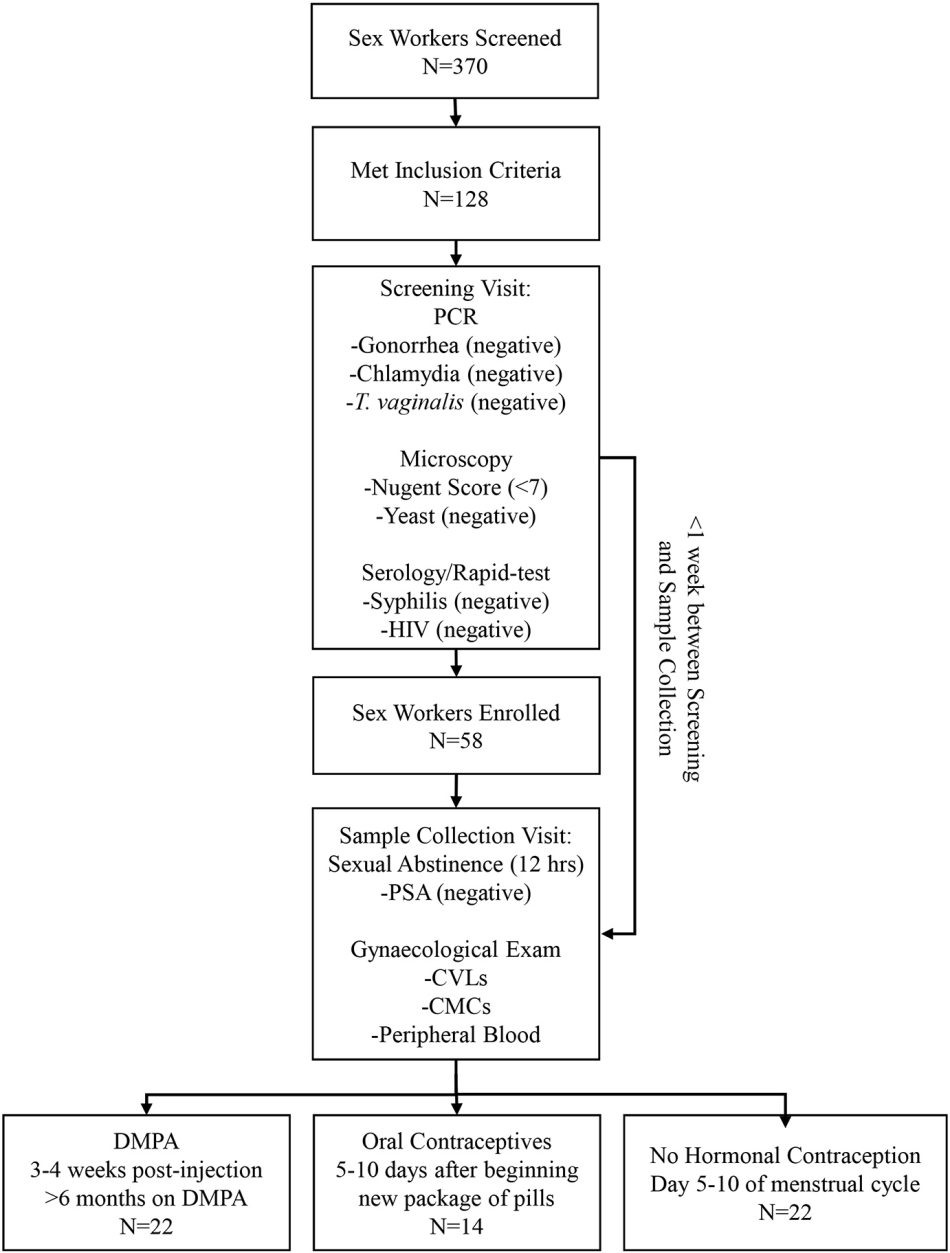
**Experimental Design.** Flow chart depicting the experimental design, screening and enrollment process for the Kenyan sex workers providing samples for the clinical study.

Our primary objective was to examine the effect of DMPA on VMB diversity. We enrolled a select group of Kenyan sex workers, according to strict inclusion/exclusion criteria (see Materials and Methods). As several factors including STIs and BV impact diversity of VMB, women with STIs and/or BV were excluded. Fifty-eight (*N*=58) women met the study criteria (Fig. 1).

**Fig. 2.**
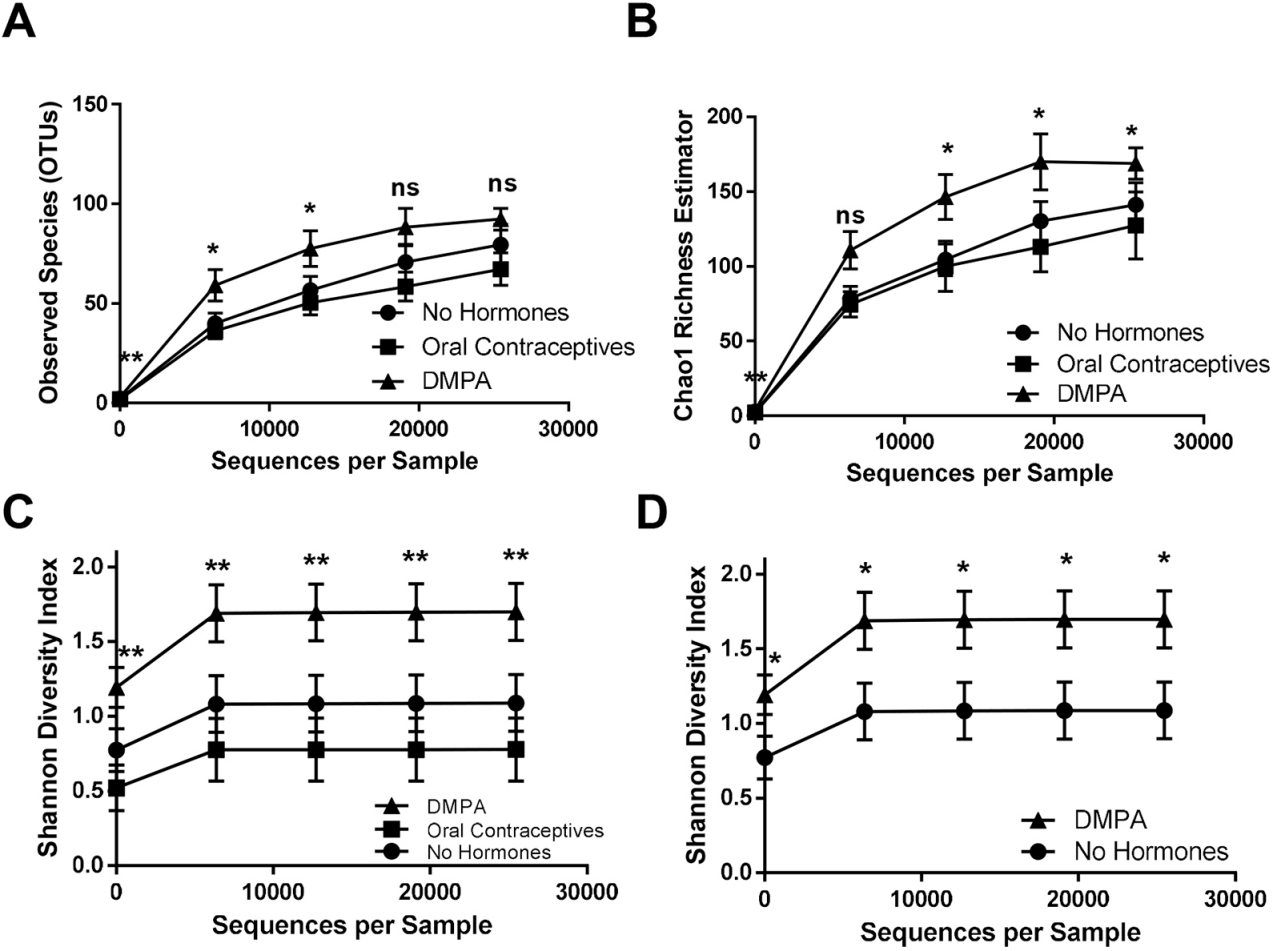
**DMPA associated with vaginal bacterial diversity in Kenyan sex workers.** (A-D) Three α-diversity metrics were used to compare bacterial richness and evenness within the vaginal microbiota of Kenyan sex workers not on hormonal contraceptives (proliferative phase of menstrual cycle, *N*=22), on oral contraceptives (*N*=14) and on DMPA (*N*=22), from the same geographical region. Significant differences in observed species were initially observed during Kruskal–Wallis tests, however no significant differences were subsequently identified during post-hoc testing (A). Chao1 richness was significantly higher in the VMB of sex workers on DMPA versus those on OCPs (*P*≤0.05; Kruskal–Wallis tests), yet no significant differences were observed between NH sex workers and those on DMPA (B). Sex workers on DMPA had the greatest bacterial diversity at all levels of rarefaction (*P*≤0.05; Kruskal–Wallis tests), followed by women not on hormonal contraceptives, and those on oral contraceptives (C). Sex workers on DMPA had significantly greater bacterial diversity in their vaginal microbiota than those not on hormonal contraceptives, at all depths of rarefaction (*P*≤0.05; Mann–Whitney *U*-test) (D). ns, not significant. *P≤0.05, **P≤0.01. Data are mean±s.e.m.

We sought to determine the effect of hormonal contraceptives on diversity of the VMB. α-Diversity was assessed in sex workers on OCPs (*N*=14, 5-10 days from beginning a new pack of pills), on DMPA (*N*=22, 3-4 weeks after last injection) and NH women (*N*=22) by observed species (richness), Chao1 (richness) and the Shannon diversity index (evenness and richness). Although significant differences in observed species (Fig. 2A) were initially observed during Kruskal–Wallis tests, no significant differences were identified during post-hoc testing. Although Chao1 richness (Fig. 2B) was significantly higher in the VMB of sex workers on DMPA versus those on OCPs, no significant differences were observed between NH women and women on DMPA. However, the VMB of sex workers on DMPA was significantly more diverse than NH (Fig. 2C,D), and OCP women (Fig. 2C), as seen in Shannon diversity rarefaction curves. To rule out that the bacterial diversity associated with DMPA might be due to a disproportionate number of women with intermediate (4-6) Nugent scores, this group was removed from the analysis and Shannon diversity was plotted in women with Nugent scores 0-3 (Fig. S1; NH, *N*=18; OCP, *N*=13; DMPA, *N*=17). Even in this subset, the Shannon diversity index was significantly greater in sex workers on DMPA than NH, suggesting that DMPA is associated with enhanced bacterial diversity in the VMB of healthy asymptomatic Kenyan sex workers. Taken together, our results suggest that DMPA is positively associated with VMB diversity in healthy asymptomatic Kenyan sex workers.

### Bacterial diversity does not correlate with vaginal inflammatory cytokines or HIV-1 target cells in healthy asymptomatic Kenyan sex workers

After determining that DMPA was associated with increased bacterial diversity in the vagina, we sought to determine whether there was a relationship between diversity of the VMB and vaginal cytokines and/or HIV-1 target cells in sex workers. Cytokines [IL-1α, IL-1RA (also known as IL1R1), IL-1β, IL-8 (CXCL8), IL-10, IFN-γ, MIP-1α (CCL3), MIP-1β (CCL4), MIG-3 (CXCL9), IP-10 (CXCL10) and MCP-1 (CCL2)] were quantified in the CVL (*N*=58) and linear regressions plotted. There were no significant correlations observed between diversity of the VMB (Shannon diversity index at 12,744 reads) and any of the aforementioned cytokines (Fig. S2).

Subsequently we sought to assess the relationship between VMB diversity and cervical HIV-1 target cells (CD4^+^CCR5^+^ T cells). There was no significant relationship between the percentage (Fig. S3A; *P*=0.496, *R*^2^=0.009) or count (N) (Fig. S3B; *P*=0.079, *R*^2^=0.06) of cervical CD4^+^CCR5^+^ T cells and VMB diversity (Shannon diversity index at 12,744 reads). A significant positive correlation was observed between VMB diversity (Shannon diversity index at 12,744 reads) and mean fluorescence intensity (MFI) of CCR5 expression on CD4^+^ T cells in the cervix (Fig. S3C; *P*=0.009, *R*^2^=0.12); however, a correlation of 0.12, in the absence of any other supporting correlation, likely does not support a biologically meaningful relationship between these two factors. Thus, overall the diversity of the VMB does not appear to correlate with cervical cytokines or HIV-1 target cells in Kenyan sex workers with Nugent scores <7.

### DMPA decreases the proportion of *Lactobacillus*-dominant VMBs in healthy, asymptomatic Kenyan sex workers

**Fig. 3.**
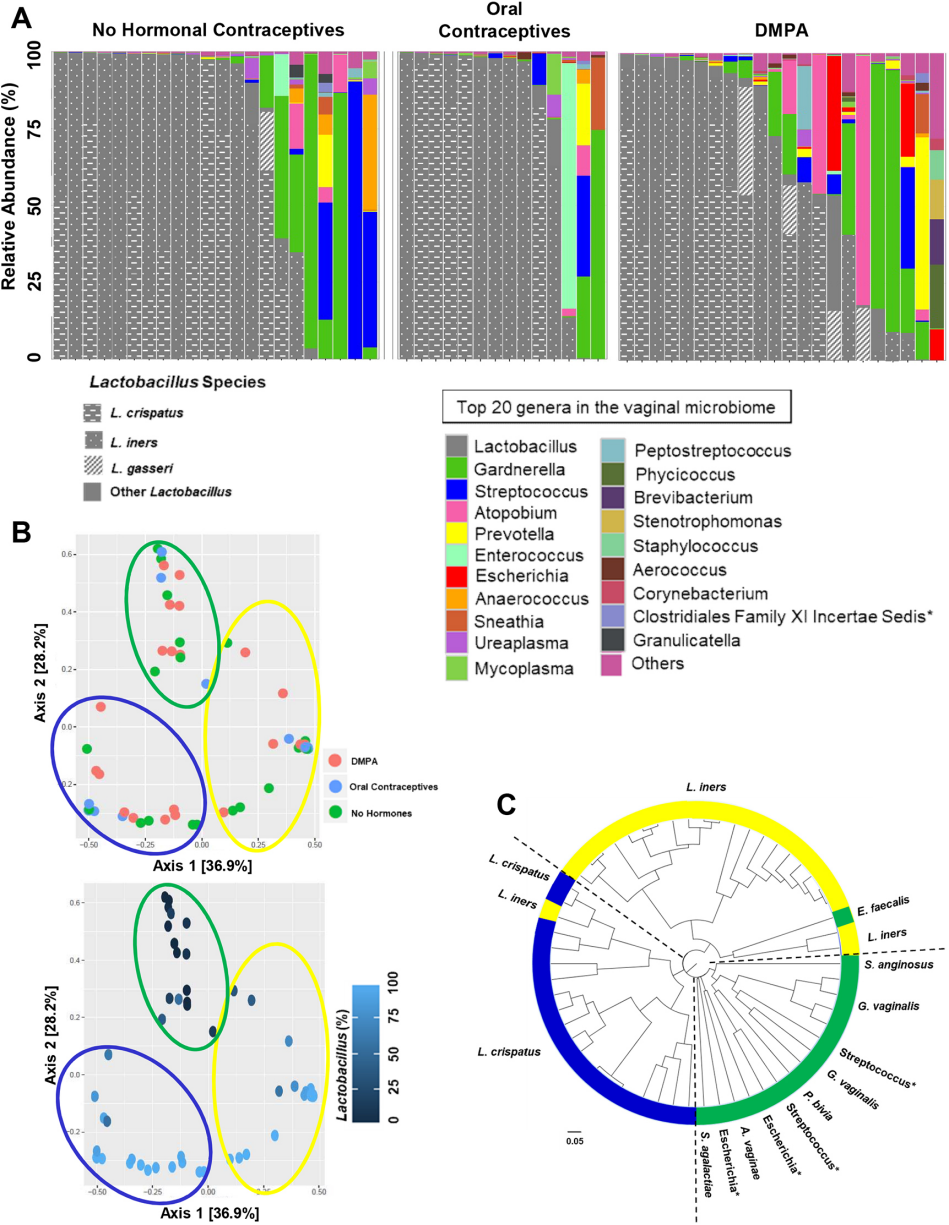
**Contraceptives and *Lactobacillus* dominance and clustering.** (A) The top 20 bacterial genera in the vaginal microbiota were plotted by relative abundance as taxa bar charts and compared between sex workers who were not on hormonal contraceptives (proliferative phase of the menstrual cycle, *N*=22), on oral contraceptives (*N*=14) or on DMPA (*N*=22). Each bar represents the vaginal microbiota of one woman. Each color represents a different genus of bacteria, as indicated. Species of *Lactobacillus* are indicated in gray/patterns. Vaginal microbiota are ordered left to right in descending order of the relative abundance of *Lactobacillus*. The proportion of women with *Lactobacillus*-dominant VMB was assessed between groups using several different cut-points of relative abundance (Table 1). At the highest cut-off chosen (≥98% lactobacilli) there were significantly more women with *Lactobacillus*-dominant VMB in the NH (proliferative phase) and OCP groups than in the DMPA group, suggesting that a high estrogen state is associated with a greater abundance of lactobacilli, and supporting our results demonstrating enhanced Shannon diversity in women on DMPA (Fig. 1C,D). Asterisk indicates resolved to family level. (B) The PCoA demonstrated the β-diversity of the vaginal microbiota at the operational taxonomic unit (OTU) level based on the Bray–Curtis dissimilarity matrix. The vaginal microbiota did not cluster by method of contraception, but clustered based on the CSTs previously described (Ravel et al., 2011), by the dominance of *Lactobacillus* in the vaginal microbiota. Sex workers dominated by *L. crispatus* (CST I) are circled in blue, those dominated by *L. iners* (CST II) in yellow, and those women with highly diverse vaginal microbiota (CST IV) are circled in green. Axes represent eigenvalues, a metric for which magnitude indicates the amount of variation captured in the PCoA axis. (C) A cluster dendrogram was calculated from Bray–Curtis dissimilarities and used to visualize clustering of the vaginal microbiota. Three clusters were observed using the gap statistic (Fig. S5A) and, in general, the previously described CSTs clustered together within the dendrogram. CST I (blue), *L. cripatus* dominant; CST II (yellow), *L. iners* dominant; CST IV (green), highly diverse. Asterisk indicates resolved to bacterial genus.

Typically, a decreased risk of HIV-1 is associated with a low diversity, *Lactobacillus*-dominant VMB (Borgdorff et al., 2014; Gosmann et al., 2017; Klatt et al., 2017). Therefore, we examined whether hormonal contraceptives affected the proportion of sex workers with *Lactobacillus*-dominant VMB. The top 20 bacterial genera were plotted by method of contraception as taxa bar charts (Fig. 3A). The type of contraceptive showed a trend, but did not significantly alter the proportion of sex workers with *Lactobacillus*-dominant VMB when we used 50-95% relative abundances as a cut-off (Table 1). However, when we selected a more stringent cut-off for *Lactobacillus* dominance (≥98% relative abundance) the type of contraceptive did significantly alter the proportion of sex workers with *Lactobacillus*-dominant VMB. There were significantly more women with *Lactobacillus*-dominant VMB in the NH (proliferative phase of menstrual cycle) and OCP groups compared with women on DMPA, suggesting that a normal/high estrogen state is associated with a greater abundance of lactobacilli, and supporting our results demonstrating enhanced Shannon diversity in women on DMPA (Fig. 2C,D).

**Table 1.**
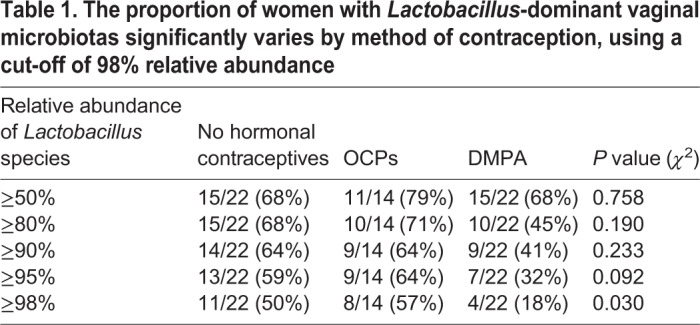
**The proportion of women with *Lactobacillus*-dominant vaginal microbiotas significantly varies by method of contraception, using a cut-off of 98% relative abundance**

In our analysis, the dominant species of *Lactobacillus* did not differ between groups (Table 2; *P*=0.737; *χ*^2^), and VMBs clustered by community state type (CST) (Ravel et al., 2011) rather than method of contraception (Fig. 3B,C) in principal coordinate analysis (PCoA) and cluster dendrograms. The gap statistic (Fig. S4A) indicated three clusters in the PCoA. PCoA ordination and Bray–Curtis dissimilarity distance were used to construct a heatmap, which also demonstrated clustering based on CSTs rather than type of hormonal contraceptive (Fig. S4B). Results suggest that, in this cohort of relatively healthy sex workers, free from BV, the proportion of women on DMPA who have highly *Lactobacillus*-dominant VMB (≥98%) is significantly decreased. In other words, these women have a greater diversity of bacteria in their VMB.

**Table 2.**
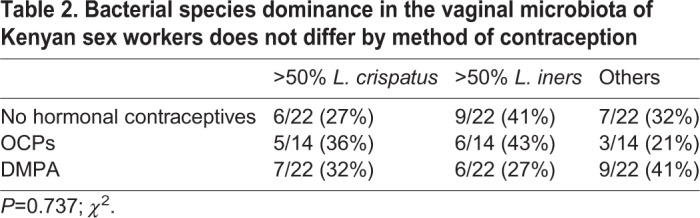
**Bacterial species dominance in the vaginal microbiota of Kenyan sex workers does not differ by method of contraception**

### Healthy asymptomatic Kenyan sex workers on DMPA have low vaginal glycogen and α-amylase

**Fig. 4.**
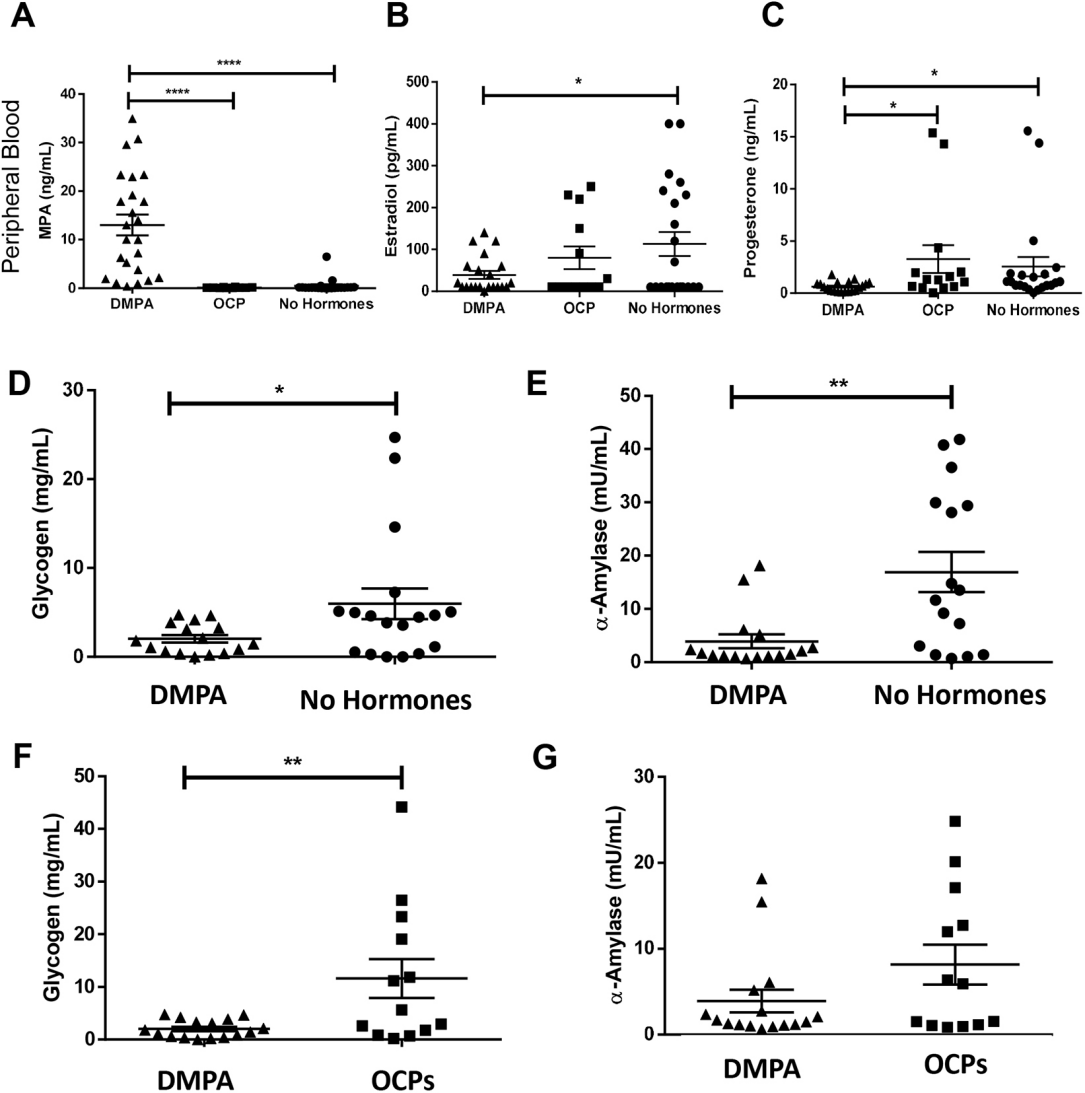
**Sex workers on DMPA have low estrogen, vaginal glycogen and α-amylase.** (A) To ensure sex workers on DMPA had detectable amounts of MPA in the circulation, plasma MPA was quantified in all study participants using ELISA. MPA was significantly higher in the sex workers in the DMPA group compared with the OCP and no hormone groups (Kruskal–Wallis test; *P*≤0.0001). (B) Sex workers on DMPA had significantly lower circulating estradiol than those not taking hormonal contraceptives (one-way ANOVA; *P*≤0.05). (C) Sex workers on DMPA also had significantly lower circulating progesterone than those on OCPs and not on hormonal contraceptives (Kruskal–Wallis test; *P*≤0.05). (D-G) As the diverse vaginal microflora can strongly influence vaginal carbohydrates in CVLs and overshadow the effect of hormones on these types of parameters (Moncla et al., 2016, 2015), we restricted our analysis of vaginal glycogen and α-amylase to sex workers with Nugent scores ≤3. (D) Vaginal glycogen was significantly lower in the vaginal lavage of sex workers on DMPA (*N*=16) compared with those not on hormonal contraceptives (proliferative phase of the menstrual cycle, *N*=18; unpaired *t*-test; *P*=0.043). (E) Similarly, α-amylase was significantly less abundant (Mann–Whitney; *P*=0.0095) in the vaginal lavage of sex workers on DMPA (*N*=16) compared with sex workers who were not on hormonal contraceptives (proliferative phase of the menstrual cycle, *N*=16). (F) Sex workers on OCPs had the highest levels of free vaginal glycogen, and significantly more free vaginal glycogen than the sex workers on DMPA (*N*=13, 16, respectively; *P*=0.008). (G) The quantity of α-amylase in the CVL supernatants of sex workers on OCPs versus those on DMPA verged on significance (*N*=13, 16, respectively; *P*=0.11). *P≤0.05, **P≤0.01, ****P≤0.0001. Data are mean±s.e.m.

Circulating MPA, estradiol and progesterone were quantified in study participants (Fig. 4A-C). As previously reported, use of DMPA was associated with hypo-estrogenism (Bahamondes et al., 2014; Hapgood et al., 2018; Miller et al., 2000); sex workers on DMPA had significantly lower levels of circulating estradiol compared with NH women (Fig. 4B). We also found that DMPA was associated with significantly lower levels of circulating progesterone compared with NH sex workers and those on OCPs (Fig. 4C).

One factor believed to promote and maintain lactobacilli in the vagina is glycogen, stored in vaginal epithelial cells and available as free glycogen in vaginal fluid (Gregoire et al., 1971; Mirmonsef et al., 2014; Nasioudis et al., 2015). Glycogen is a glucose polymer converted to disaccharides by α-amylase, and can be used by lactobacilli and other bacteria (Macklaim et al., 2013) as an energy source. Estrogen has been proposed to enhance glycogen in the human vaginal epithelium (Cruickshank and Sharman, 1934; Farage and Maibach, 2006), and glycogen is experimentally enhanced in the vaginal tissues of estrogen-treated hamsters and non-human primates (Gregoire and Parakkal, 1972; Gregoire and Richardson, 1970). As DMPA is potently anti-estrogenic (Hapgood et al., 2018), we examined whether DMPA was associated with lower levels of free glycogen and α-amylase in the vaginal microenvironment compared with NH women (with endogenous hormones) or those on estrogen-containing OCPs. As others have demonstrated that the diverse vaginal microflora can overshadow the effect of hormones on vaginal glycosidases and lectins (Moncla et al., 2016, 2015), we restricted this analysis to women with Nugent scores ≤3. Vaginal glycogen [Fig. 4D; *N*=16 (DMPA), 18 (NH); *P*=0.043] and α-amylase [Fig. 4E; *N*=16 (DMPA), 16 (NH); *P*=0.0095] were significantly less abundant in the CVL supernatants of sex workers on DMPA compared with NH women (2.04±0.42 versus 5.98±1.72 mg/ml; and 3.91±1.32 versus 16.90±3.78 mU/ml, respectively). Furthermore, sex workers on estrogen-containing OCPs had the highest free vaginal glycogen, and significantly more vaginal glycogen than women on DMPA [Fig. 4F; *N*=16 (DMPA), 13 (OCPs); *P*=0.008], supporting the concept that estrogen is linked with enhanced vaginal glycogen. No significant difference in α-amylase was observed between women on DMPA and OCPs [Fig. 4G; *N*=16 (DMPA), 13 (OCPs); *P*=0.1066].

As glycogen is believed to maintain lactobacilli in the vagina, we assessed the relationship between free glycogen and *Lactobacillus* abundance. Free glycogen in the CVL positively correlated with abundance of *Lactobacillus* species in the VMB (Fig. S5A; *N*=54; linear regression; *P*=0.046, *R*^2^=0.07), while the relationship between α-amylase and *Lactobacillus* verged on significance (Fig. S5B; *N*=48; linear regression; *P*=0.148, *R*^2^=0.04). In addition, we observed a negative correlation between bacterial diversity and vaginal glycogen (Fig. S5C; *N*=54; *P*=0.0015, *R*^2^=0.18), and a negative correlation between bacterial diversity and α-amylase (Fig. S5D; *N*=55; *P*=0.05, *R*^2^=0.07). Together, results suggest DMPA has a negative impact on vaginal glycogen and α-amylase in sex workers, two factors thought to be important for vaginal colonization by lactobacilli (Gregoire et al., 1971; Mirmonsef et al., 2014, 2015; Nasioudis et al., 2015).

### DMPA is associated with low vaginal glycogen in humanized mice

**Fig. 5.**
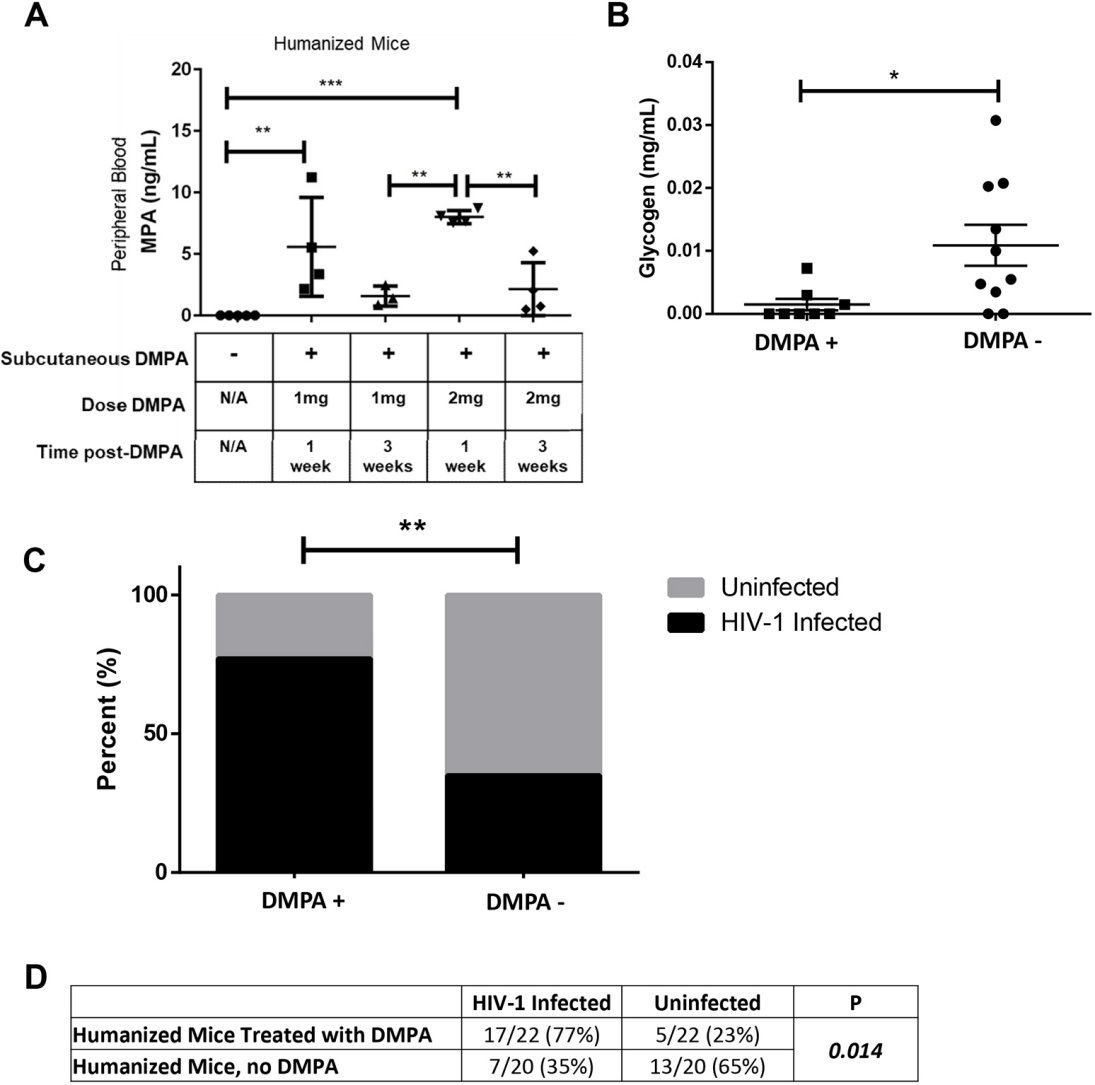
**DMPA is associated with lower vaginal glycogen and increased HIV-1 susceptibility in humanized mice.** (A) To determine whether a subcutaneous injection of DMPA in the nape of the mouse neck could induce circulating concentrations similar to those observed in women, we administered 1 (*N*=7) or 2 mg (*N*=8) of DMPA or saline (control, *N*=5) to NRG mice (background strain of the humanized mice), collected peripheral blood by cardiac puncture 1 or 3 weeks later, and quantified MPA in the serum using ELISA. (B) Glycogen was quantified in the vaginal homogenate of humanized mice (controls, no hormonal treatment, *N*=10) and those treated with DMPA (*N*=8). Vaginal glycogen was significantly lower in the humanized mice treated with DMPA (Mann–Whitney *U*-test; *P*=0.017). (C) Control humanized mice (no hormonal treatment, *N*=20) and humanized mice given 2 mg DMPA (*N*=22) were challenged intravaginally with HIV-1. The infection rate in DMPA-treated humanized mice (77%) was higher than that in untreated control humanized mice (35%) (*χ*^2^; *P*=0.014). (D) The proportion of DMPA-treated humanized mice that became infected with HIV-1 was significantly greater (*χ*^2^; *P*=0.014) than the proportion of untreated control humanized mice that became infected following intravaginal challenge. N/A, not applicable. *P≤0.05, **P≤0.01, ***P≤0.001. Data are mean±s.e.m.

Given that it is difficult to mechanistically assess the effect of DMPA on the vaginal microenvironment and susceptibility to HIV-1 in humans, we sought to determine whether similar effects were seen in an experimentally controlled system. We recently optimized a humanized mouse model, reconstituted with human immune cells, that demonstrates HIV-1 infection following intravaginal challenge (humanized NRG mice; Nguyen et al., 2017). To ensure subcutaneous injection of DMPA could induce circulating concentrations similar to those observed in women, we administered 1 or 2 mg of DMPA or saline to NRG mice, and collected blood 1 or 3 weeks later. MPA was quantified in duplicate in serum (Fig. 5A). We used the 2 mg dose in subsequent experiments because variability in circulating MPA was lower at 1 week compared with the 1 mg dose, and because a similar peak and plateau phase occurred to that seen in women (Hapgood et al., 2018).

As estradiol is thought to enhance glycogen in vaginal epithelium, and DMPA is hypo-estrogenic, we posited we would see similar effects of DMPA on glycogen in humanized mice, as we had seen in the Kenyan sex worker cohort. Glycogen was quantified in vaginal homogenates from humanized mice that had received DMPA or not (no hormonal treatment). Vaginal glycogen was significantly lower in uninfected DMPA-treated humanized mice compared with uninfected untreated control mice [Fig. 5B; 1.5×10^−3^±9.0×10^−4^ versus 1.1×10^−2^±3.0×10^−3^ mg/ml; *N*=10 (untreated), 8 (DMPA-treated); *P*=0.017]. Thus, DMPA suppresses vaginal glycogen in humanized mice, similar to the correlation we observed in sex workers.

### DMPA enhances susceptibility to HIV-1 in humanized mice

DMPA is associated with increased susceptibility to HIV-1 in women (Polis et al., 2016), and women with high diversity VMB are more susceptible to HIV-1 (Gosmann et al., 2017). Here, we demonstrate that DMPA is associated with VMB diversity in a Kenyan sex worker cohort, which may contribute to increased HIV-1 risk. No studies have demonstrated a direct link between DMPA and enhanced susceptibility to HIV-1 in humanized mice. We thus experimentally assessed whether DMPA-treated humanized mice were more susceptible to HIV-1 in a heterosexual model of viral transmission (Nguyen et al., 2017) compared with untreated, control humanized mice. Humanized mice (control, no hormonal treatment, *N*=20) and DMPA-treated humanized mice (*N*=22) were challenged intravaginally with 10^5^ TCID50/ml NL4.3-Bal-Env HIV-1 (Nguyen et al., 2017; Platt et al., 2009). HIV-1 viral load in peripheral blood was quantified by clinical RT-PCR 3-5 weeks following challenge (Nguyen et al., 2017). The proportion of infected DMPA-treated humanized mice was significantly higher (17/22, 77%, *P*=0.014) than those not receiving DMPA (7/20, 35%) (Fig. 5C,D). These results show that in an experimental model, treatment with DMPA significantly enhanced HIV-1 infection following intravaginal viral exposure.

## DISCUSSION

**Fig. 6.**
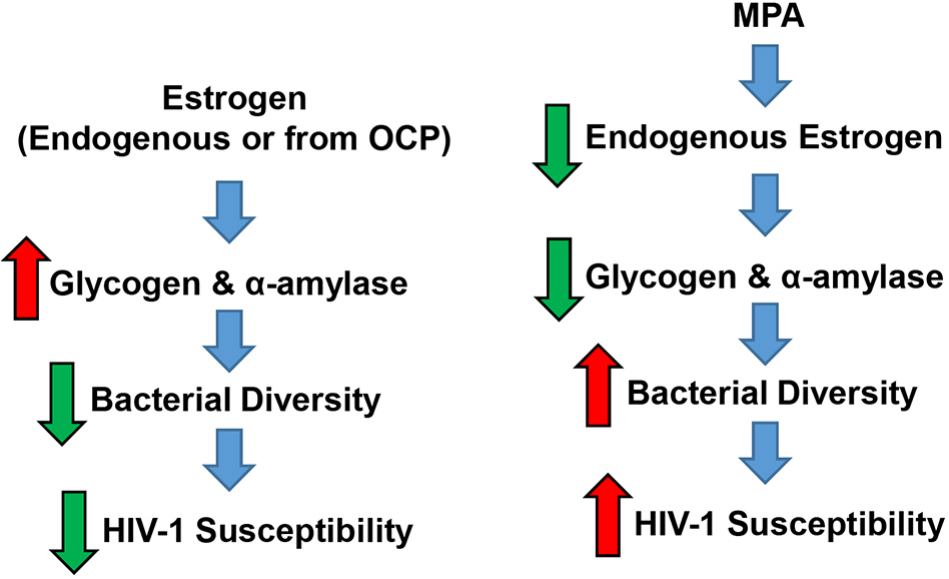
**Proposed mechanism linking DMPA to changes in vaginal microenvironment and HIV-1 susceptibility in Kenyan sex workers.** Taken together, our results suggest that estrogen (either from endogenous sources or oral contraceptive pills) is associated with greater quantities of vaginal glycogen and α-amylase, and minimal bacterial diversity within the vaginal microbiota of sex workers. This stable, uniform microbiota is not associated with susceptibility to HIV-1, albeit via incompletely understood mechanisms. In contrast, the hypo-estrogenism resulting from use of MPA lowers key metabolic products (i.e. glycogen, and α-amylase) that can be used by certain protective bacterial species, and the change in substrates allows other bacteria to colonize the vaginal microbiota, in effect increasing bacterial diversity. Diversity of the vaginal microbiota may contribute to enhanced susceptibility to HIV-1 in sex workers by an unknown mechanism that is independent of inflammatory cytokines and/or enhanced T cells.

This study provides compelling clinical evidence linking vaginal bacterial diversity to use of the hormonal contraceptive DMPA in healthy asymptomatic Kenyan sex workers. We also demonstrate that plasma estradiol, vaginal glycogen and α-amylase are low in sex workers on DMPA. We recapitulated some of these results in an experimental model, in which DMPA-treated humanized mice had less vaginal glycogen and enhanced susceptibility to HIV-1 following intravaginal challenge. Based on these results we posit a potential model linking DMPA-induced hypo-estrogenism to changes in the VMB and microenvironment that might impact susceptibility to HIV-1 (Fig. 6). However, the mechanism does not appear to include upregulation of inflammatory cytokines nor activated T cells. These results represent progress towards understanding the biological link between MPA, the vaginal microenvironment and susceptibility to HIV-1 in sex workers without clinical BV.

Low diversity *Lactobacillus*-dominated VMB are thought to protect against HIV-1 in women (Gosmann et al., 2017; Nunn et al., 2015). Lactobacilli appear to provide non-specific defense against a broad range of pathogens via production of lactic acid, hydrogen peroxide and anti-microbial bacteriocins, and by providing a physical/neutralizing barrier that inhibits other bacteria/pathogens (Hapgood et al., 2018). The current understanding of the relationship between vaginal bacteria and HIV-1 susceptibility stems mostly from the BV literature. Women with Nugent scores ≥7 are diagnosed with clinical BV. Historically, BV is correlated with increased HIV-1 risk (Atashili et al., 2008; Cohen et al., 2012; Low et al., 2011). In early studies using high-resolution sequencing, women with BV were also seen to have polymicrobial microbiotas, which were associated with inflammation and increased HIV-1 target cells (Masson et al., 2015; Thurman et al., 2015). Thus, many studies considered BV to be equivalent to high bacterial diversity. However, it is now becoming clear that the two do not necessarily overlap (McKinnon et al., 2019; Wessels et al., 2017), and a subset of women with high VMB diversity measured using 16S rRNA gene sequencing do not have clinical BV (as assessed by Nugent scoring). It is also increasingly clear that bacterial diversity, even in the absence of clinical BV, might confer greater susceptibility to HIV-1 (Gosmann et al., 2017; Low et al., 2011), as women with intermediate vaginal flora still had a 1.5× increased risk of HIV-1 acquisition in a meta-analysis (Low et al., 2011). Here, we showed that MPA is associated with VMB diversity in Kenyan sex workers with low Nugent scores, without BV. Although we and others (Jespers et al., 2017) have seen this association using 16S rRNA gene sequencing in women with Nugent scores <7, it is important to note that hormonal contraceptives (DMPA and OCPs) are typically associated with reduced BV, as assessed by Nugent scoring (van de Wijgert et al., 2013). However, Nugent scoring is based on microscopic visualization of the abundance of three bacterial morphotypes, and is not meant to assess bacterial diversity as compared with the α-diversity metrics calculated by 16S rRNA gene sequencing. As BV overshadows the effect of hormones on vaginal biomarkers of microbial health (glycosidases, lectins) in CVLs (Moncla et al., 2016, 2015), and because BV is already associated with increased risk of HIV-1 (Atashili et al., 2008), we chose to exclude women with Nugent scores 7-10 and focus on understanding how DMPA and diversity of the VMB in the absence of BV might influence HIV-1 susceptibility in Kenyan sex workers.

We also found lower vaginal glycogen and α-amylase in Kenyan sex workers on DMPA. Glycogen is a glucose polymer that can be converted to disaccharides by α-amylase and used by bacteria such as lactobacilli as an energy source (Macklaim et al., 2013). These two factors are thought to select for a *Lactobacillus-*dominated microbiota in the human vagina, which is protective against many STIs (Gregoire et al., 1971; Mirmonsef et al., 2014, 2015; Nasioudis et al., 2015). Estrogen is believed to enhance vaginal glycogen (Cruickshank and Sharman, 1934; Farage and Maibach, 2006; Gregoire and Parakkal, 1972; Gregoire and Richardson, 1970) via incompletely understood mechanisms. Thus, a normal/high estrogen and glycogen state, as was observed in NH and OCP women, would be expected to lead to a greater abundance of lactobacilli, which metabolize glycogen. When we used a cut-off of ≥98% relative abundance of lactobacilli, this association was apparent (Table 1). This suggests that the hypo-estrogenism associated with DMPA (Fig. 4B) might change carbohydrate resources in the vagina and negatively impact protective bacteria, allowing other species not as reliant on glycogen to colonize, and thus enhance bacterial diversity. This may be particularly true in a sex worker cohort, where women are more likely to be exposed to a variety of bacteria because they likely have more sex, multiple partners and different sexual practices than women who are not sex workers. Importantly, MPA was also associated with low vaginal glycogen in humanized mice. This suggests that, regardless of host species, MPA affects carbohydrate resources within the vaginal microenvironment, and bacterial species able to access the vagina and thrive under the new conditions respond accordingly.

Although DMPA is associated with increased bacterial diversity, low vaginal glycogen and α-amylase in the vagina of Kenyan sex workers, we wanted to determine whether MPA enhanced HIV-1 susceptibility in humanized mice. We challenged humanized mice intravaginally with HIV-1, and DMPA-treated mice were more likely to become infected than controls (77% versus 35%, *P*=0.014). This is the first report of DMPA enhancing HIV-1 infection in humanized mice, and this model represents a novel platform for discovery, development and testing of interventions to mitigate the effect of DMPA on HIV-1 susceptibility, which will ultimately move this field of research forward. We had hypothesized that diversity of the VMB would be associated with enhanced inflammatory cytokines and/or T cells, ultimately enhancing susceptibility to HIV-1 because activated cervical HIV-1 target cells (CD4^+^CCR5^+^CD25^+^) were increased in women on long-term progestin-only contraceptives (DMPA, norethisterone enanthate) (Byrne et al., 2016), and a 17-fold increase in activated cervical target cells (CD4^+^CCR5^+^CD38^+^HLA^–^DR^+^) in women with high diversity VMB was reported (Gosmann et al., 2017). Furthermore, women with high diversity VMB have elevated inflammatory cytokines (IL-1β, IL-1ɑ and IL-8) (Masson et al., 2014), and vaginal bacteria affect inflammatory responses and barrier function in the female reproductive tract (Anahtar et al., 2015; Birse et al., 2017; Doerflinger et al., 2014; Lennard et al., 2017). There is also evidence that DMPA enhances susceptibility to simian immunodeficiency virus in non-human primates via inflammatory pathways (Li et al., 2009). However, we did not observe altered inflammatory cytokines (IL-1α, IL-1RA, IL-1β, IL-8, IL-10, IFN-γ, MIP-1α, MIP-1β, MIG-3, IP-10 or MCP-1) or T cells in Kenyan sex workers on DMPA with Nugent scores <7, suggesting that the mechanism by which the vaginal microbiota may contribute to HIV-1 susceptibility in this cohort is independent of these factors. It is important to note that we did not assess other HIV-1 target cells (dendritic cells, macrophages), and cervical inflammation in sex workers is dampened (Lajoie et al., 2014; McLaren et al., 2010). We also did not assess the association between bacterial diversity and integrity of the vaginal epithelium, which could also impact susceptibility to HIV-1.

We are not the only group to examine the effect of DMPA on VMB diversity. Birse et al. (2017) found no significant association between DMPA and bacterial diversity (Shannon diversity index) in non-sex workers, however women with BV were not excluded (Birse et al., 2017). The results of the present study support those of Jespers et al. (2017), who demonstrated that injectable progestins (including DMPA) were associated with VMB diversity and reduced concentration of lactobacilli in African women (Jespers et al., 2017). Although another study included non-sex workers of varying ethnicities, and included BV, bacterial diversity (by inverse Simpson's index) was significantly lower in women on OCPs than DMPA or not on hormonal contraceptives, and although not statistically significant, women on DMPA had greater bacterial diversity than women not on hormonal contraceptives (Brooks et al., 2017). These studies support the association between MPA and VMB diversity in healthy, asymptomatic Kenyan sex workers that was observed in our study.

Our study was not without limitations. The CVLs in this study were briefly centrifuged at a low speed to pellet out cellular debris following their collection, and profiling of the VMB was performed on the CVL supernatant, as opposed to the cell pellet. This was based on the assumption that the bacteria detectable in the CVL obtained from the vaginal lumen reflect the overall composition of the VMB, whereas the cell pellets may be enriched for adherent bacteria. A recent study in macaques, in which different sampling methods were compared (vaginal swab versus CVL cell pellet versus CVL supernatant), found that all three methods yielded comparable profiles of the VMB (Schmidt et al., 2019). However, because our assays were performed on the CVL supernatant and not the cell-associated pellet, it is possible that we may have missed certain adherent bacterial species or communities present in the VMB owing to these technical limitations. In addition, factors including ethnicity, cultural background and vaginal douching affect the VMB (Borgdorff et al., 2014; Chaban et al., 2014; Eschenbach et al., 2000; Gajer et al., 2012; Ravel et al., 2011; Schwebke et al., 1999; Srinivasan et al., 2012). We therefore recruited sex workers of the same ethnicity and geographical region to minimize confounders. Although we collected information on vaginal douching, we do not have information on intravaginal drying practices, which are associated with VMB diversity (Birse et al., 2017). However, because we did not see a significant difference in the proportion of sex workers practicing vaginal douching, we do not anticipate a difference in women performing vaginal drying, categorized by method of contraception. One limitation of our clinical study was the inability to mechanistically examine how DMPA and the associated changes in the vaginal microbiota and microenvironment might impact susceptibility to HIV-1. To compensate, we experimentally recapitulated some of our clinical study results in a controlled manner in humanized mice, and importantly demonstrated that DMPA-treated humanized mice are indeed more susceptible to HIV-1 than untreated mice. In the present study, we collected samples from NH women on days 5-10 of the menstrual cycle to minimize cycle variability and standardize cycle phase. However, this phase is the estrogen high phase of the menstrual cycle, and because we did not recruit sex workers during the progesterone high phase, one could question whether differences in diversity were due to sample collection time for NH women. However two independent prospective studies have shown the Shannon diversity index remains stable across the menstrual cycle (Chaban et al., 2014; Gajer et al., 2012). We thus believe results represent an accurate depiction of the effect of MPA on VMB diversity and the vaginal microenvironment in sex workers, especially given results of recent studies (Brooks et al., 2017; Jespers et al., 2017).

The vagina represents a major site of HIV-1 acquisition in women, and use of DMPA is consistently associated with enhanced HIV-1 susceptibility. It is interesting to note that a recent large trial that compared three different popular contraceptives, DMPA, copper intrauterine device and levonorgestrel implant, did not report any significant differences in HIV incidence among the three contraceptives users, although it found very high rates of HIV among all three groups (Evidence for Contraceptive Options and HIV Outcomes Trial Consortium, 2019). Sex workers are among priority populations because their risk for HIV-1 is 50× higher than women not involved in sex work. Understanding the effects of MPA on the VMB of sex workers is therefore an important public health issue. This is especially true considering DMPA is used by 8 million women in sub-Saharan Africa (Ross and Agwanda, 2012), where HIV-1 is endemic. Here, we demonstrate that MPA is associated with decreased estradiol levels in plasma and increased VMB diversity, as well as suppression of vaginal glycogen and α-amylase in Kenyan sex workers, factors believed to promote vaginal colonization by protective bacteria. We also show that MPA has similar effects on vaginal glycogen in humanized mice, and enhances susceptibility to HIV-1 following intravaginal challenge. We thus propose that one of the mechanisms by which MPA increases susceptibility to HIV-1 in sex workers is via suppressing endogenous estrogen, which may be responsible for maintaining glycogen and α-amylase. As a result of the change in substrates, other species not as reliant on these substrates access and compete for space and colonize the vagina. The subsequent increase in bacterial diversity enhances susceptibility to HIV-1 in these sex workers, likely via a non-inflammatory mechanism(s), such as disruption of the vaginal epithelial barrier function. Future studies aimed at exploring MPA-mediated mechanisms of HIV-1 susceptibility in women, especially non-sex workers, and humanized mice are warranted.

## MATERIALS AND METHODS

### Study approval and experimental design

This study was approved by McMaster University (HiREB 0332-T) (AUP# 14-09-40), University of Manitoba (B2015:033) and Nairobi/Kenyatta National Hospital (KNH-ERC P132/03/2015). Our objectives were to determine whether DMPA affected VMB diversity, the proportion of *Lactobacillus-*dominant sex workers, VMB clustering, and vaginal glycogen and α-amylase. Some of our results were replicated in humanized mice, where objectives were to experimentally confirm that DMPA affected vaginal glycogen and susceptibility to HIV-1.

Study participants were screened/enrolled through the Sex Workers Outreach Programme (SWOP), Pumwani Sex Worker cohort in Nairobi, Kenya (Fowke et al., 1996). Women were recruited from January 2015 to April 2016, following the study outline (Fig. 1) and the strict inclusion/exclusion criteria below. Written informed consent and demographic information (Table 3) were received from all participants prior to inclusion in the study, following explanation of the study and potential risks.

**Table 3.**
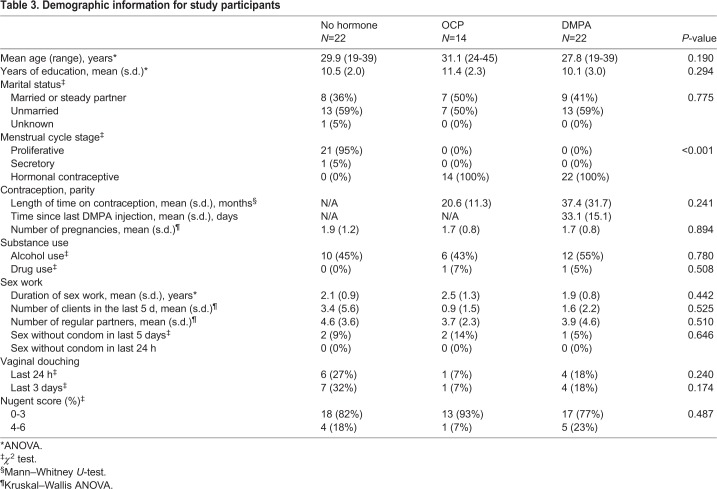
**Demographic information for study participants**

Women were included if >18 years old, pre-menopausal, willing to undergo pelvic exams, intact uterus and cervix, in good health and negative for STIs (gonorrhea, chlamydia, *Trichomonas vaginalis*, syphilis, HIV), negative for yeast infection, Nugent score <7 at screening, could abstain from douching for 24 h and sexual activities for 12 h before sample collection, use condoms for 36 h prior to abstinence, and had not been in sex work >5 years. Sex workers on DMPA were using injectable DMPA >6 months and screening was within 3-4 weeks of the most recent injection. Sex workers on OCPs were taking OCPs containing estrogen and progesterone (recorded/confirmed by staff during screening) >6 months and screening was within 5-10 days of beginning a new pack. Sex workers not using hormonal contraceptives (NH) were not taking any hormonal contraception for >6 months and screening was within 5-10 days of beginning a menstrual cycle (proliferative phase). MPA was quantified in duplicate in the plasma of all women using ELISA (EuroProxima), as per the manufacturer's protocol, following modifications (Smith et al., 2014) (Fig. 4A). Plasma concentrations of estradiol and progesterone were quantified for all women using the MILLIPLEX MAP Steroid/Thyroid Hormone Magnetic Bead Panel (Millipore, Merck) and following the manufacturer's instructions (Fig. 4B,C). Women were excluded if they were bleeding or spotting at the time of sample collection, pregnant (or had been within 1 year), breastfeeding, unwilling to provide consent or follow protocol, using progesterone-only OCPs or did not meet strict inclusion criteria.

At screening, urine, blood, and vaginal swabs were collected. Urine was tested for *Neisseria gonorrhoeae*, and *Chlamydia* species by PCR (Xpert CT/NG kits, Cepheid AB). Blood was collected for syphilis and HIV serology for all participants using a rapid test (Determine, Inverness Medical), and HIV serostatus was confirmed using ELISA (Vironostika, bioMérieux Clinical Diagnostics). Each woman underwent a gynaecological exam to obtain vaginal specimens for microscopy (Nugent score, yeast infection and *Trichomonas vaginalis*). Women positive for STI(s) were excluded and treated according to Kenyan protocols. HIV+ women were referred for anti-retroviral therapy.

Sample collection occurred within 1 week following screening. A prostate specific antigen (PSA) test (Seratec PSA Semiquant) was performed to confirm 12 h abstinence, as recent unprotected sex could alter VMB. A gynecological exam was performed and endocervix washed with 2 ml sterile 1× phosphate buffered saline (PBS) from a 3 ml aliquot. CVL was collected from the posterior vaginal fornix, placed in a sterile tube on ice and sent to the laboratory, where it was centrifuged at low speed (120 ***g***) to remove cellular debris. Supernatants and remaining 1 ml PBS (negative controls) were aliquoted in a biosafety cabinet, frozen and stored at −80°C until shipped in liquid nitrogen to Winnipeg, Manitoba, Canada. CVLs and negative controls were shipped on dry ice to Hamilton, Ontario, Canada, for VMB analysis, and α-amylase and glycogen quantification. A cervical cytobrush to isolate cervical mononuclear cells (CMC) was collected following CVL and immediately sent to the University of Nairobi, Kenya, for flow cytometry.

### Bacterial V3 region of 16S rRNA gene sequencing

Genomic DNA was extracted from the CVL supernatants using a modified DNA isolation method as described in Stearns et al. (2015). CVL supernatants were inverted to mix, and transferred to screw-cap tubes containing 2.8 mm ceramic beads, 0.1 mm glass beads, guanidine EDTA sarcosine and sodium phosphate buffer. Samples were bead beaten and centrifuged as described in Stearns et al. (2015), and the resulting supernatant was further processed using the robotic MagMAX Express 96-Deep Well Magnetic Particle Processor (Applied Biosystems) with the Multi-Sample kit (Life Technologies, 4413022).

Purified DNA was used to amplify the V3 region of the 16S rRNA gene by PCR. DNA (50-100 ng) was used as a template with 1 U of Taq, 1× PCR Buffer (Life Technologies), 1.5 mM MgCl_2_, 0.4 mg/ml bovine serum albumin, 0.2 mM dNTPs and 5 pmols each of Illumina adapted primers 341F (CCTACGGGAGGCAGCAG) and 518R (ATTACCGCGGCTGCTGG) (primers+Illumina adapters/barcode/priming region as described in supplemental materials of Bartram et al., 2011: ∼80 bp) (Bartram et al., 2011; Wessels et al., 2017; Whelan and Surette, 2017). The PCR reaction was carried out at 94°C for 5 min, 30 cycles of 94°C for 30 s, 50°C for 30 s and 72°C for 30 s, with a final extension of 72°C for 10 min. Any resulting PCR products were visualized on a 1.5% agarose gel. Positive amplicons (visualization of 16S band on the agarose gel) were normalized using the SequalPrep normalization kit (Thermo Fisher Scientific, A1051001) and sequenced on the Illumina MiSeq platform at the McMaster Genomics Facility (McMaster University, Canada). Resulting sequences were run through the sl1p pipeline as previously described (Whelan and Surette, 2017).

Negative controls were: (1) DNA extraction controls (all reagents, but no CVL supernatants) were included in the robotic processing and subsequent PCR to ensure resulting bacterial profiles were not due to kit contaminants. (2) Each PCR run contained no template negative controls, which did not yield PCR products (no 300 bp band on agarose gel). (3) Four 1 ml aliquots of PBS (negative controls) were randomly selected and processed as above, alongside CVL supernatants for gDNA extraction and PCR amplification of the 16S rRNA gene. These negative controls did not yield PCR products (no 16S bands were visualized on agarose gels). As per the McMaster Genomics Facility protocol, samples not yielding a PCR product for the 16S rRNA gene were not sent for sequencing. These samples were considered to be negative. We have thus considered bacterial contamination during CVL collection, handling, processing, extraction, and PCR to be minimal/negligible in our samples.

### Cytokine quantification

CVL supernatant cytokine concentrations were quantified using the Milliplex MAP kit (Millipore) and analyzed using BioPlex-200 (Bio-Rad). CVL supernatants were incubated overnight, and analytes quantified included IL-1α, IL-1RA, IL-1β, IL-8, IL-10, IFN-γ, MIP-1α, MIP-1β, MIG-3, IP-10 and MCP-1.

### Glycogen and α-amylase quantification

Free glycogen in CVL supernatants was quantified colorimetrically using the Glycogen Assay Kit (BioVision) (Mirmonsef et al., 2014). CVL supernatants were thawed on ice, vortexed and spun. The CVL supernatant (5 µl in quadruplicate) was added to a 96-well plate and the volume adjusted to 50 µl with hydrolysis buffer. The hydrolysis enzyme was added to two wells/sample; those without enzyme were negative controls (background glucose in CVL supernatants). Negative controls were subtracted from final values to determine total free glycogen in CVL supernatants. Optical densities were read using a SpectraMax i3 (Molecular Devices). A pancreatic human amylase ELISA (Abcam) (Spear et al., 2014) was used to quantify α-amylase in CVL supernatants (1:1 dilution, kit diluent). The assay sensitivity was 4.0×10^−4^ mg/ml (BioVision).

### Flow cytometry

CMC pellets were immunophenotyped at the University of Nairobi. Cells were washed with FACS buffer and stained for 30 min at 4°C with ECD-Live-Dead (Invitrogen), then washed twice with FACS buffer. Cells were suspended in blocking solution (mouse IgG, FACS buffer, fetal bovine serum) for 10 min at 4°C and washed with FACS buffer. A cocktail of antibodies and Brilliant Violet Stain (all from BD Biosciences) was used to stain cells for 30 min at 4°C. The antibodies used were: CD3 PeCY5 (clone HIT3a, 1/2), CD4 Alexa 700 (clone RPA-T4, 1/4), CD8 APCH7 (clone SK1, neat), CCR6 BB515 (clone 11A9, 1/8), HLA-DR BV510 (clone G46-6, neat), CD69 PeCy7 (clone FN50, 1/5), BV421 (clone 2D7/CCR5, 1/2), CD161 APC (clone DC12, 1/2) and CD38 PE (clone HIT2, neat). Cells were washed and fixed in 1% paraformaldehyde. Data were acquired using a LSRII flow cytometer (BD Biosciences) and analyzed using FlowJo v10.0.8r1 (TreeStar).

### Humanized mice

Experimental protocols were approved by HiREB and McMaster University Animal Research Ethics Board (AREB), AUP# 14-09-40, in accordance with Canadian Council of Animal Care guidelines. Humanized NRG mice were generated as described (Nguyen et al., 2017). Briefly, 4-day-old mice were irradiated and injected with CD34-enriched placental cord blood stem cells and left for 12 weeks to allow human immune reconstitution of bone marrow and peripheral tissues.

### Humanized mouse hormone treatments

Humanized mice (*N*=4) were anaesthetized and 21-day slow-release (476 ng/mouse/day) estradiol pellets (Innovative Research of America, USA) were implanted in the neck (Anipindi et al., 2016). The quantity of estradiol induced is similar to the estrous cycle (Modder et al., 2004). To ensure that subcutaneous injection of DMPA could induce circulating concentrations similar to those seen in humans, we administered 1 mg (*N*=7) or 2 mg (*N*=8) of DMPA or saline (control, *N*=5) to NRG mice, and collected blood by cardiac puncture 1 or 3 weeks later. MPA was quantified in duplicate in serum using ELISA (EuroProxima), as above (Fig. 5A). We used the 2 mg dose in subsequent experiments because variability in circulating MPA was lower at 1 week, compared with the 1 mg dose, and because a similar peak and plateau phase occurred to that seen in women (Hapgood et al., 2018).

### Glycogen quantification in the humanized mouse vagina

Free glycogen was quantified in vaginal tissue of uninfected humanized mice (*N*=10). Mice were treated with 2 mg DMPA for 1 or 4 weeks (*N*=8, 4/time) as above and vaginal homogenates were boiled at 100°C for 10 min to inactivate enzymes, according to the Glycogen Assay Kit (BioVision) protocol. Homogenates (10 µl in quadruplicate) were added to a 96-well plate and assay performed as above.

### Intravaginal HIV-1 challenge in humanized mice

Control (*N*=20) and 2 mg DMPA-treated (*N*=22) humanized mice were challenged intravaginally with 10^5^ TCID50/ml NL4.3-Bal-Env HIV-1 (Nguyen et al., 2017). Humanized mice were HIV-1+ if viral load was detected in peripheral blood by clinical RT-PCR, at week 3 or 5 post-infection (timepoints selected for their ability to reliably detect HIV-1 in the blood) (Nguyen et al., 2017).

### Statistical analysis

16S rRNA sequences were processed by our in-house data pipeline (Wessels et al., 2017; Whelan and Surette, 2017). α-Diversity including singletons was calculated using a sl1p pipeline (Whelan and Surette, 2017), with QIIME version 1.7.0-dev. Ten rarefaction tables with 67,821 sequences were used. Observed species, Chao1 and Shannon diversity were graphed and analyzed using GraphPad Prism (GraphPad Software). Data are presented as mean±s.e.m. Taxa bar charts, Bray–Curtis dissimilarity PCoAs, heatmaps, cluster analyses, cluster dendrograms and species estimations were generated as described (Wessels et al., 2017).

Categorical variables were compared using Fisher's Exact Test or *χ*^2^ (SigmaStat 3.5 Systat Software). Continuous variables were compared by Student's *t*-test, Mann–Whitney Rank Sum Test, one-way ANOVA or Kruskal–Wallis tests (Graphpad Software). *P*≤0.05 was considered significant.

## Supplementary Material

Supplementary information
