# Supplementary material for: Medroxyprogesterone acetate alters the vaginal microbiota and microenvironment in women and increases susceptibility to HIV-1 in humanized mice
Source: Dis Model Mech. 2019 Oct 23;12(10):dmm039669. doi: 10.1242/dmm.039669 (PMC6826019; doi:10.1242/dmm.039669)
Supplement: Supplementary information [file dmm-12-039669-s1.pdf]

Disease Models & Mechanisms • Supplementary information

Supplemental Figure

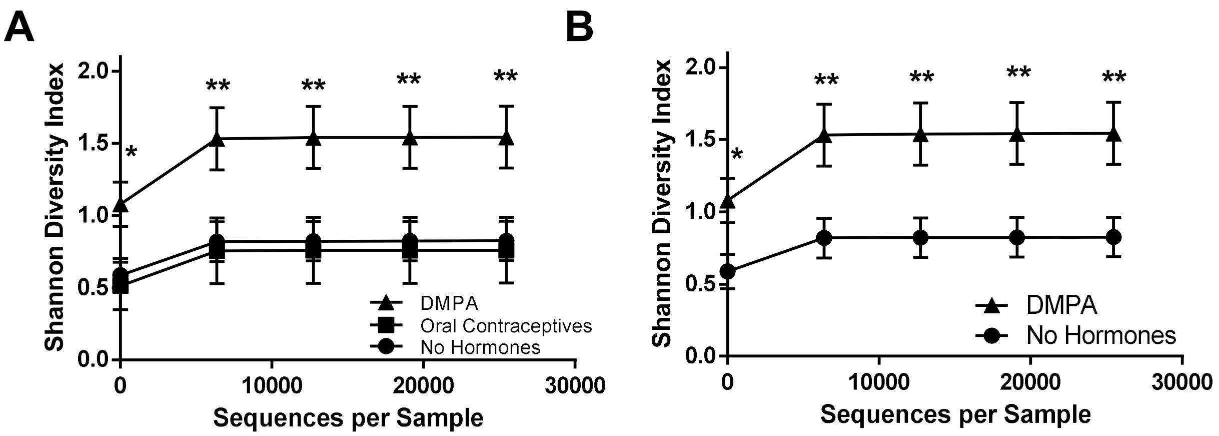

Figure S1: DMPA Associated with Vaginal Bacterial Diversity in Sex Workers with Nugent Score ≤3.

The Shannon Diversity Index was plotted in sex workers with Nugent Scores 0-3, to ensure the enhanced bacterial diversity we observed in the CVLs of sex workers on DMPA was not due to women with Nugent Scores 4-6. (**A**) Sex workers with Nugent Scores 0-3 who were on DMPA (N=17) had the greatest bacterial diversity followed by those with Nugent Scores 0-3 who were not on hormonal contraceptives (proliferative phase of the menstrual cycle, N=18), and those with Nugent Scores 0-3 on oral contraceptives (N=13), and at all levels of rarefaction in Shannon Diversity plots (P≤0.05; Kruskal-Wallis tests). (**B**) Sex workers with Nugent Scores 0-3 who were on DMPA had significantly greater bacterial diversity in their vaginal microbiota than those with Nugent Scores 0-3 who were not on hormonal contraceptives (proliferative phase of the menstrual cycle), at all depths of rarefaction (Mann-Whitney U tests; P≤0.05). Even in this subset of sex workers with Nugent Scores of 0-3, the Shannon Diversity Index was significantly greater in sex workers on DMPA than those not on hormonal contraceptives. DMPA: Depot-medroxyprogesterone acetate. *: P≤0.05. **: P≤0.01. Data is presented as mean ± SEM.

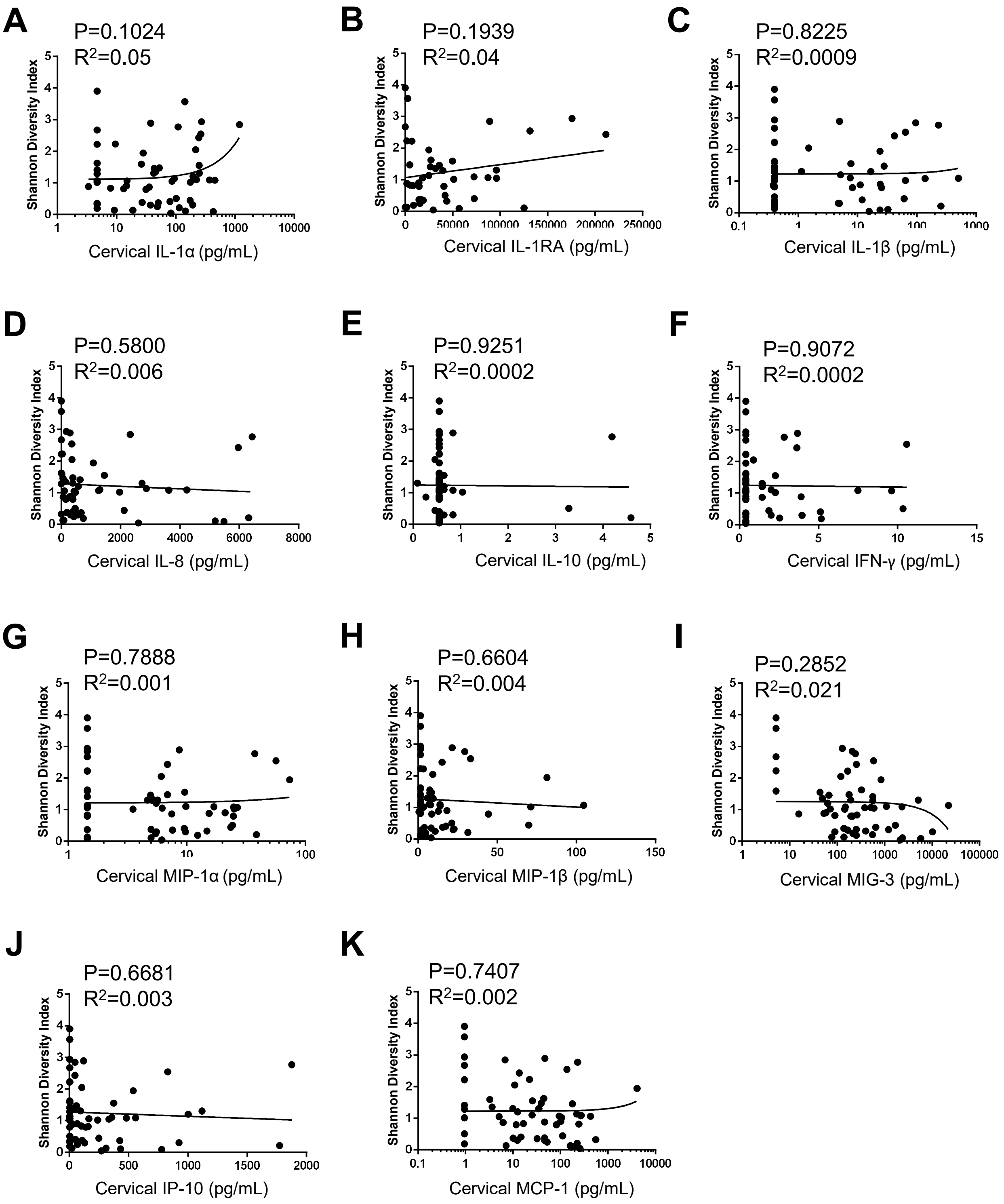

Disease Models & Mechanisms • Supplementary information

Figure S2: Diversity of the Vaginal Microbiota and Cervical Cytokines in Sex Workers.

Linear regression analyses were performed between diversity of the vaginal microbiota (Shannon Diversity Index at 12744 reads) and cervical cytokines to determine if diversity of the vaginal microbiota correlated with factors that might impact susceptibility to HIV-1 in Kenyan sex workers (N=58). No significant relationship between the quantity of cervical **(A)** IL-1α, **(B)** IL-1RA, **(C)** IL-1β, **(D)** IL-8, **(E)** IL-10, **(F)** IFN-γ, **(G)** MIP-1α, **(H)** MIP-1β, **(I)** MIG-3, **(J)** IP-10, or **(K)** MCP-1 and VMB diversity (Shannon Diversity Index at 12744 reads) was observed.

Disease Models & Mechanisms • Supplementary information

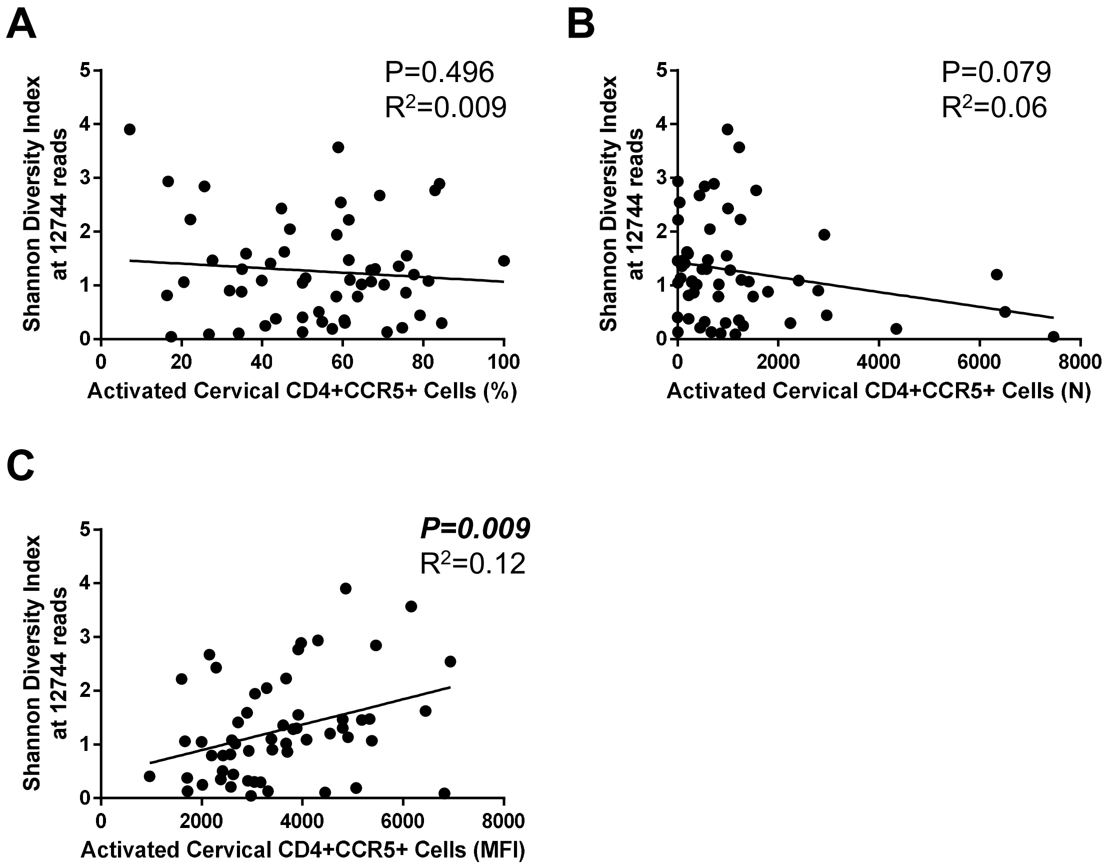

Figure S3: Diversity of the Vaginal Microbiota and HIV-1 Target Cells in Sex Workers.

Linear regression analyses were performed between diversity of the vaginal microbiota (Shannon Diversity Index at 12744 reads) and HIV-1 target cells (CD4+CCR5+) to determine if diversity of the vaginal microbiota correlated with factors that might impact susceptibility to HIV-1 in Kenyan sex workers (N=58). No significant relationship between the percentage **(A)** of cervical CD4+CCR5+ T cells and VMB diversity (Shannon Diversity Index at 12744 reads) was observed (P=0.496, R^2=0.009), and no significant relationship between the number (count (N)) **(B)** of cervical CD4+CCR5+ T cells and VMB diversity (Shannon Diversity Index at 12744 reads) (P=0.079, R^2=0.06) was observed. However, **(C)** a significant positive correlation was observed between VMB diversity (Shannon Diversity Index at 12744 reads) and activation status (MFI) of CD4+CCR5+ T cells in the cervix (P=0.009, R^2=0.12), suggesting that with increasing bacterial diversity there may be increased CCR5 receptors on the HIV-1 target cells (CD4+ cells) present in the cervix. However, the R^2 value is low, and thus it is difficult to discern if this is a biologically meaningful association. MFI: mean fluorescence intensity.

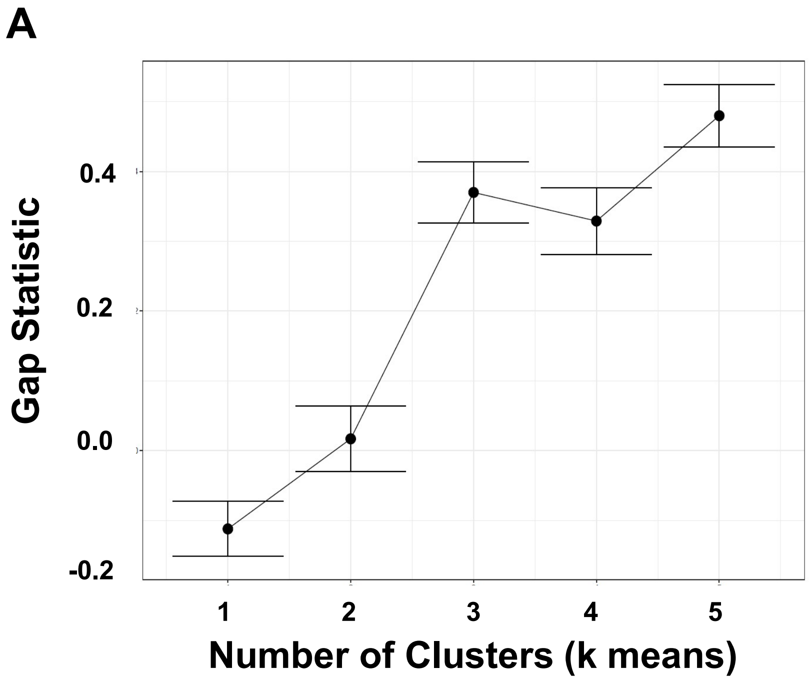

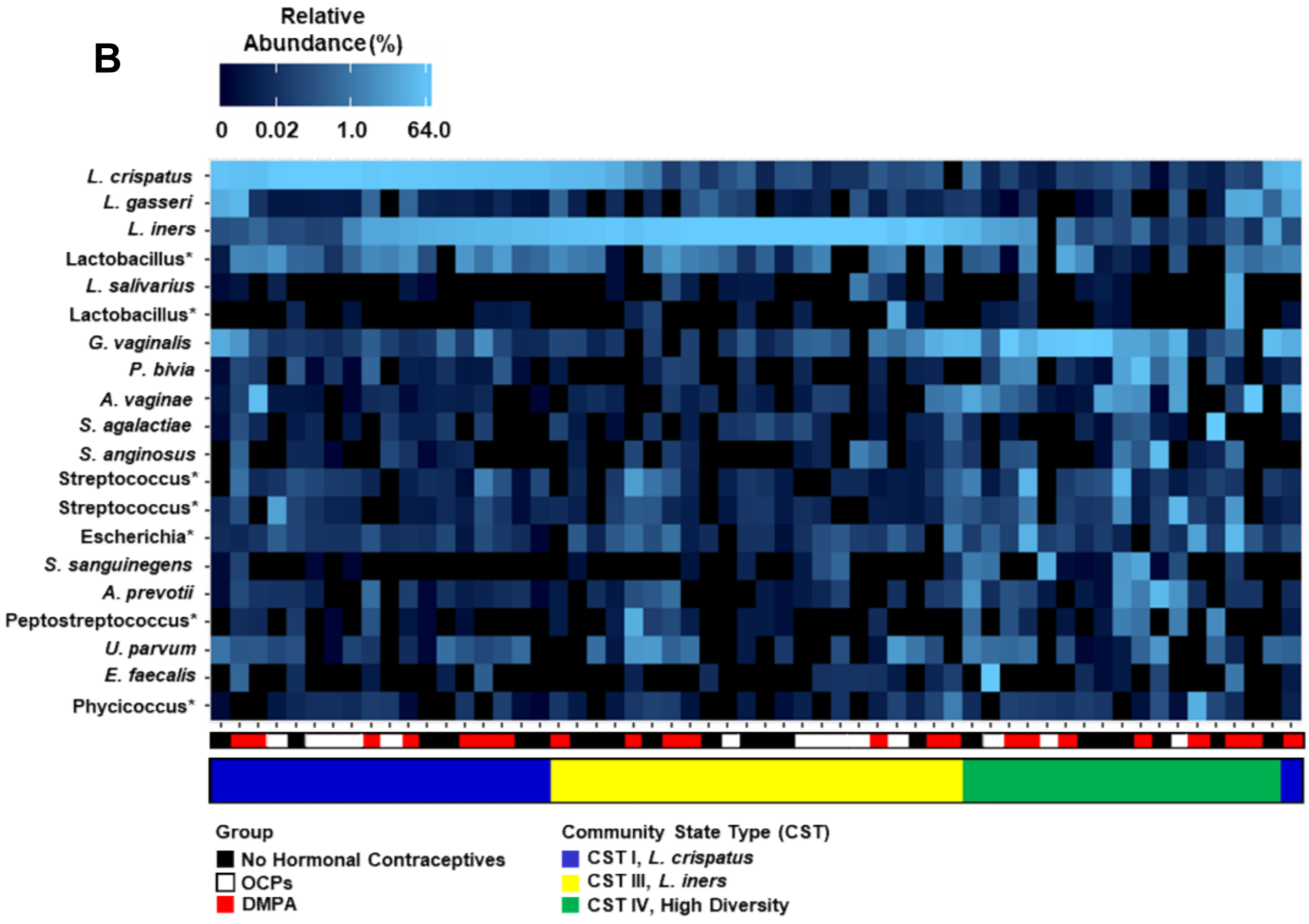

Disease Models & Mechanisms • Supplementary information

Figure S4: Clustering of the Vaginal Microbiota in Sex Workers.

(A) The gap statistic, which gives an estimation of how well 'k' (number of clusters) fits the data in the PCoA plot, was calculated. The number of clusters present in the data is indicated by the plateau in the gap statistic, which occurred at 3. **(B)** A heatmap of the top 20 species based on Bray-Curtis dissimilarity distance and PCoA ordination revealed that the vaginal microbiota (columns) did not cluster based on hormonal contraceptive type, but clustered by community state type (CST). Clustering of the vaginal microbiota by relative abundance of lactobacilli was also observed in the heatmap. Taxa are ordered along the y-axis by ranked order of abundance. CST: Community State Type. DMPA: Depot-medroxyprogesterone acetate. OCP: Oral contraceptive pills. *: resolved to bacterial genus.

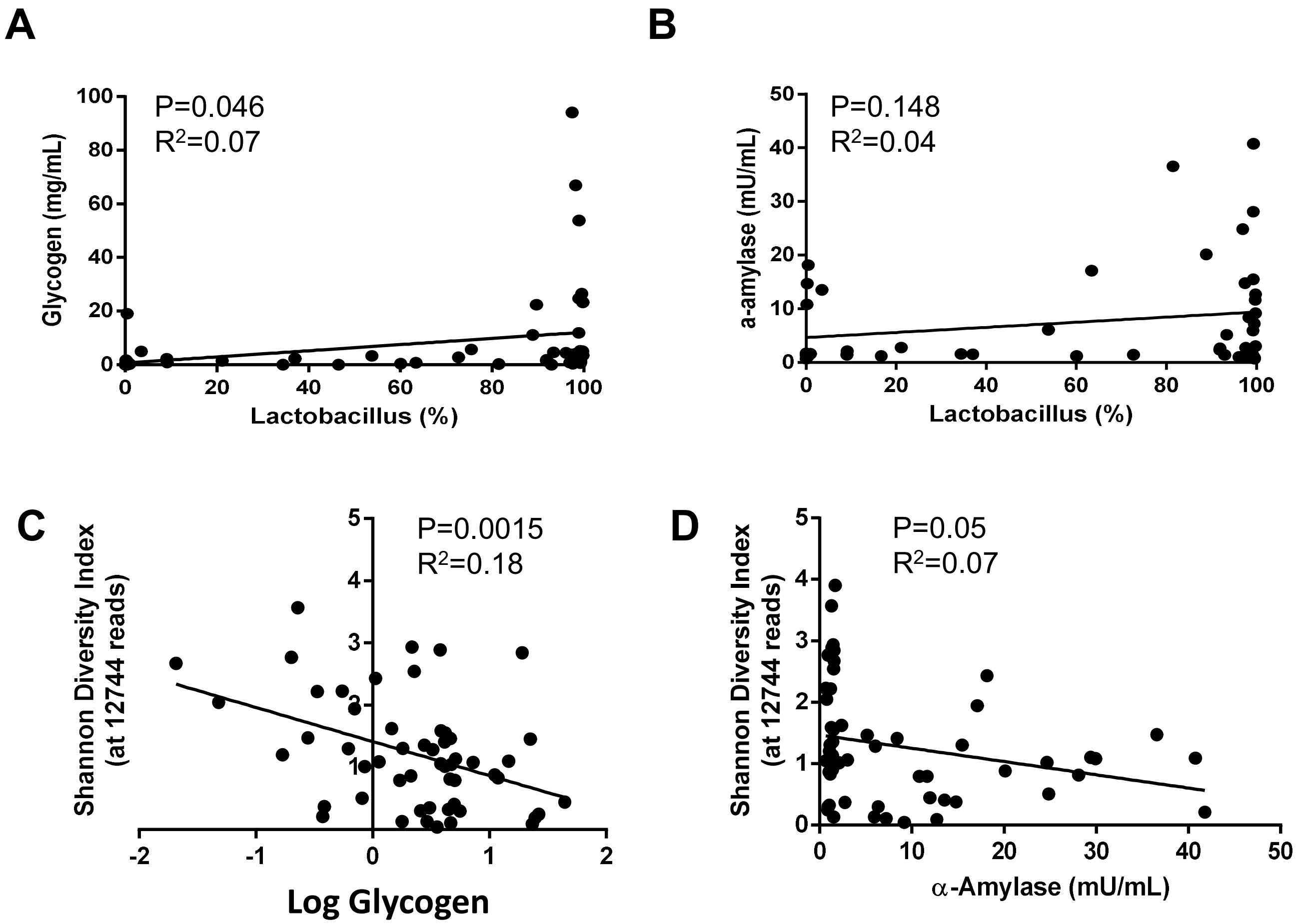

Figure S5: Relationships between Glycogen, α-Amylase, Lactobacilli, and Bacterial Diversity.

(A) There was a positive correlation between free glycogen in the vaginal lavage and abundance of *Lactobacillus* species in the vaginal microbiota (N=54; Linear Regression; P=0.046, R^2=0.07). **(B)** The relationship between α-amylase in the vaginal lavage and abundance of *Lactobacillus* species in the vaginal microbiota verged on significance (N=48; Linear Regression; P=0.148, R^2=0.04). **(C)** A negative correlation between diversity of the vaginal microbiota (Shannon Diversity Index) and glycogen in the vaginal lavage was observed (N=54; Linear Regression; P=0.0015, R^2=0.18). **(D)** There was also a negative correlation between bacterial diversity and α-amylase in the vaginal lavage (N=55; Linear Regression; P=0.05, R^2=0.07). However, the R^2 values are low, and thus it is difficult to discern if these are biologically meaningful associations. mU/mL: milli units per millilitre.

Disease Models & Mechanisms • Supplementary information
